# Marine Algae Incorporated Polylactide Acid Patch: Novel Candidate for Targeting Osteosarcoma Cells without Impairing the Osteoblastic Proliferation

**DOI:** 10.3390/polym13060847

**Published:** 2021-03-10

**Authors:** Salih Veziroglu, Mustafa Ayna, Theresa Kohlhaas, Selin Sayin, Jacek Fiutowski, Yogendra Kumar Mishra, Fatih Karayürek, Hendrik Naujokat, Eyüp Ilker Saygili, Yahya Açil, Jörg Wiltfang, Franz Faupel, Oral Cenk Aktas, Aydin Gülses

**Affiliations:** 1 Chair for Multicomponent Materials, Institute for Materials Science, Faculty of Engineering, Kiel University, Kaiserstr. 2, 24143 Kiel, Germany; sve@tf.uni-kiel.de (S.V.); ff@tf.uni-kiel.de (F.F.); 2 Department of Periodontology, University Hospital of Bonn, D-53111 Bonn, Germany; mayna@uni-bonn.de; 3 Department of Oral and Maxillofacial Surgery, University Hospital of Schleswig-Holstein, Campus Kiel, Arnold-Heller-Straße 3, 24105 Kiel, Germany; tete@theresakohlhaas.de (T.K.); Hendrik.Naujokat@uksh.de (H.N.); Yahya.Acil@uksh.de (Y.A.); Joerg.Wiltfang@uksh.de (J.W.); 4 Marine Science and Technology Faculty, Iskenderun Technical University, 31200 Iskenderun, Turkey; selin.sayin@iste.edu.tr; 5 Mads Clausen Institute, NanoSYD, University of Southern Denmark, Alsion 2, 6400 Sønderborg, Denmark; fiutowski@mci.sdu.dk (J.F.); mishra@mci.sdu.dk (Y.K.M.); 6 Department of Periodontology, Cankiri Karatekin University, 18100 Cankiri, Turkey; fatihkarayurek@karatekin.edu.tr; 7 Department of Medical Biochemistry, SANKO University, Şehitkamil, 27090 Gaziantep, Turkey; ISaygili@sanko.edu.tr

**Keywords:** composite patch, marine algae, osteoblast, sarcoma, sargassum vulgare

## Abstract

Biodegradable collagen-based materials have been preferred as scaffolds and grafts for diverse clinical applications in density and orthopedy. Besides the advantages of using such bio-originated materials, the use of collagen matrices increases the risk of infection transmission through the cells or the tissues of the graft/scaffold. In addition, such collagen-based solutions are not counted as economically feasible approaches due to their high production cost. In recent years, incorporation of marine algae in synthetic polymers has been considered as an alternative method for preparation grafts/scaffolds since they represent abundant and cheap source of potential biopolymers. Current work aims to propose a novel composite patch prepared by blending Sargassum vulgare powders (SVP) to polylactide (PLA) as an alternative to the porcine-derived membranes. SVP-PLA composite patches were produced by using a modified solvent casting method. Following detailed material characterization to assess the cytocompatibility, human osteoblasts (HOBs) and osteosarcoma cells (SaOS-2) were seeded on neat PLA and SVP-PLA patches. MTT and BrdU assays indicated a greater cytocompatibility and higher proliferation for HOBs cultured on SVP-PLA composite than for those cultured on neat PLA. SaOS-2 cells cultured on SVP-PLA exhibited a significant decrease in cell proliferation. The composite patch described herein exhibits an antiproliferative effect against SaOS-2 cells without impairing HOBs’ adhesion and proliferation.

## 1. Introduction

Many patients suffer from critical bone defects secondary to trauma, diseases, ablative tumor surgeries or congenital malformations [1], which necessitate bone grafting procedures and bone tissue engineering solutions, including scaffolds, membranes, and patches. Especially in bone tissue engineering procedures, polymers are one of the most preferred materials, thanks to their wide range of application scopes. Basically, polymers can be classified into three groups: entirely synthetic polymers, natural polymers, and their combinations [2]. Synthetic polymers allow for better control of chemical, physical, and mechanical properties, as well as degradation rate [3]. In addition, the flexibility in fabrication and processing methods allow the production of synthetic polymers in a wide range of forms with desired porosity, morphology, and anisotropy, which are crucial factors for the enhancement of cell attachment and proliferation. However, possible toxicity and inflammatory responses of synthetic polymers have also been reported by various research groups [4].

Aliphatic polyesters such as polyglycolic acid (PGA), polyhydroxyalkanoate (PHA), polycaprolactone (PCL), polylactic acid (PLA), and their copolymers are most preferred polymers in bone tissue engineering [5]. Among these polymers, PLA receives the most attention owing to its easy processability, nontoxicity, and moderate biocompatibility. [6] PLA has been used in various biomedical products, including sutures, braces, bone screws, wound dressing and bandages [7]. Besides that, PLA could be also used as “loaded scaffolds” containing bioactive molecules [8] or incorporated with connective tissue proteins to promote tissue regeneration [9]. In addition to their use in biomedical applications, they are also extensively preferred as packaging and film material. Therefore, the blend of PLA with a synthetic or a natural polymer (rather than its neat form) should also fulfil some thermal and mechanical requirements [10].

Biocomposite patches, prepared by combining the synthetic PLA matrix with algae-based fillers, are shown to exhibit superior thermal and mechanical properties [11]. In recent years, various studies proclaimed that the use of marine algae, which are abundant in the coastal areas of various countries, as filler material improves the mechanical properties of synthetic polymers and also reduces the overall production cost [12,13]. In addition to their usage as simple and cost-effective fillers, various studies have shown that marine algae have a high nutrient content and exhibit several biologically active components which could ensue multibiological properties [14]. For example, while glycoproteins in *Porphyra yezoensis* (red algae) are known to inhibit the proliferation of tumour cells, polyose found in *Kappaphycus alvarezii* (red algae) are reported to enhance the immunity [15]. Moreover, amino acids and fatty acids in *Monostroma angicava* (green algae) and *Spirogyra sp.* (green algae) are shown to prevent thrombosis and help prevent circulatory system diseases, respectively [16,17].

A brown algae type *Sargassum vulgare (S. vulgare)* attracts the attention of various research groups since it is a potential source of a wide spectrum of bioactive materials, including alginate, fucans, sulfated polysaccharide, and polyphenols [18]. Additionally, for several decades, it has been well known that the alginates extracted from *S. vulgare* exhibit a considerable antitumoral activity [19,20,21]. For instance, the antiangiogenic activity and antitumor effect of *S. vulgare* towards the myeloblastic leukemic cells and cervical cancer cells has been reported [22]. An ideal antitumoral material should enable a peri-operative attack on tumor cells without impairing the cytocompatibility or proliferation of healthy cells of the tumor environment [23]. In addition to its proven bioactivity against bacteria, viruses, and other pathogens, the proliferative effect of *S. vulgare* on some cell types has been also reported by several researchers [24,25]. Recently, it has been proclaimed that the *S. vulgare*-PLA composites could induce fibroblast proliferation and might be a feasible option in clinical practice such as guided tissue regeneration, connective tissue augmentation, and wound regeneration [26].

The problems such as inadequate treatment due to the severe adverse effects of the chemotherapeutic drugs and as well as the inability to reach therapeutic concentrations at the tumour site are still challenging. Considering these clinical problems, the composite patch described herein may be helpful for delivering a direct multistage attack on the tumour cells and/or to reduce the risk of malignant transformations. Additionally, it could be hypothesized that, the incorporation of a bio-originated material into the mentioned composite patch could provide superior biocompatibility as wells as cost-effectiveness. Therefore, the current work aims to propose a novel composite patch prepared by blending PLA with *S. vulgare* powders (SVP), which exhibits an antiproliferative effect against osteosarcoma cells without impairing osteoblast adhesion and proliferation.

## 2. Materials and Methods

### 2.1. Preparation of SVP

The *S. vulgare* samples were collected near Iskenderun, Turkey from the Mediterranean Sea at a depth of 25–40 m in May 2020.Following the visual analysis and identification with the help of stereomicroscope (Olympus SZX10, Hamburg, Germany), *S. vulgare* samples were immersed in 1000 mL of distilled water for 72 h to remove any water-soluble components. After filtering, samples were dried at 50–60 °C for 24 h under vacuum (Memmert V0101, Memmert GmbH, Schwabach, Germany). Dried samples were subsequently ground four times in a high-speed rotary grinder operating (Retsch RM 200, Retsch GmbH, Duesseldorf, Germany) at 250 rpm for 15 min (every round) and air-dried at 40–50 °C for 24 h. At the last stage, dried samples were once more ground and passed through 300- and 400-mesh sieves, air-dried for one day at 50–60 °C, and vacuum-dried for at least 12 h at 100–110 °C until the absolute moisture content of the resulting fine, the brownish powder was 3 ± 0.5%. Biochemical composition of macro-algae extracts contained; ash, 16.31 ± 0.16%, lipids, 10.27 ± 0.42% and proteins, 7.20 ± 0.18%. The traditional conversion factor was set to 6.25 [27]. A rapid method for total lipid extraction and purification [28].

### 2.2. Preparation of SVP-PLA Composite

**Figure 1:**
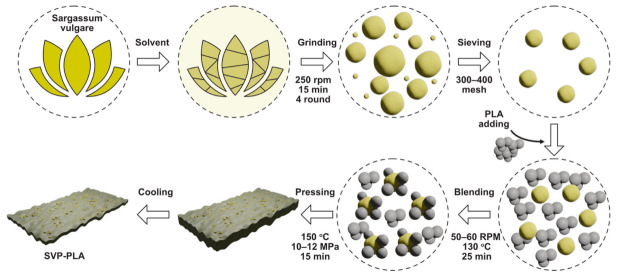
Schematic illustration of the *S. vulgare* powder-poly-lactic acid (SVP-PLA) composite preparation.

SVPs were washed with acetone and dried in an oven at 90–100 °C for 24 h. Then dried SVPs (20 wt.%) were mixed with PLA (80 wt.%) (Goodfellow, 459-898-81, Coraopolis, PA, USA) in a mechanical mixer operating at 150 °C for 25 min at a rotor speed of 50–60 rpm [29]. After mixing, the composites were poured into a Teflon mold and pressed into thin plates with a custom hot press operating at 150 °C and 10–12 MPa for 15 min. Afterwards, the hot-pressed composite was cooled down to RT in the oven. The whole process is schematically depicted in Figure 1. Composite patches were cut into 1 cm × 1 cm sample dimensions for further characterization.

### 2.3. Preparation of PLA Patches

Neat PLA patches were also prepared by applying the similar process given above (except adding SVP) and then cut into 10 mm× 10 mm samples.

### 2.4. Material Characterization

A digital camera (Panasonic GH5S, Osaka, Japan) was used to reveal the macromorphological properties of prepared neat PLA and SVP-PLA patches. Orion NanoFab-Carl Zeiss helium-ion microscope (HIM) (Oberkochen, Germany) was used to investigate the surface morphology of the prepared PLA-SVP composite. Fourier-transform infrared (FTIR) spectra of dry samples have been acquired with Bruker Vertex 80v spectrometer (Bruker, Billerica, MA, USA) operating in the range of 1000 cm^−1^ and 3500 cm^−1^. The baseline correction was done using built-in software.

### 2.5. Cytocompatibility Analysis

All experiments were performed in triplicate at the 6, 24, and 72 h after incubation. Human osteoblast cells (HOBs) were obtained from the cancellous bone samples of crista iliaca anterior of patients who had undergone bone graft harvesting for augmentative procedures of the maxillofacial region at the Department of Oral and Maxillofacial Surgery at the University Hospital Schleswig-Holstein, Campus Kiel. Preoperatively, all patients were informed about the sampling process, and a written consent was obtained. The bone samples were transferred into the culture medium (89% Dulbecco’s modified Eagle´s minimum essential medium (DMEM) PAA Laboratories, Austria, 10% fetal calf serum (FCS; Biochrom, Germany, 1% Penicillin/Streptomycin, Biochrom, Germany). Further processing has been done under laminar flow in a biological safety cabinet (Heraeus Instruments, Osterode, Germany). The samples were minced into 1 mm pieces and placed into cell culture flasks (T- 300, Thermo-Fisher Scientific, Waltham, MA, USA) containing 10 mL of the respective culture medium. Cell culture medium was changed every three to four days. The passaging of both cell groups was performed when they reach the 80% confluence. Briefly, the medium has been aspirated, rinsed with 10 mL of phosphate buffered solution (PBS, Sigma, St. Louis, MO, USA), 5 mL of PBS containing 0.05% trypsin were added per culture flask for deattachment of cells from the surface. Cell suspension was diluted with DMEM containing 10% FCS to inhibit the action of trypsin. The cell suspension was centrifuged at 3200 rounds per minute (rpm) for 3 min at 400x g force (Eppendorf, H.amburg, Germany). The supernatant was suctioned off, and the remaining cell pellet was resuspended in 5 mL of medium. Subsequently, the cells were counted in a Neubauer counting chamber (Brand, Wertheim, Germany). In each case, 10^5^ cells were transferred into a new 75 cm^3^ culture flask containing 10 mL of medium. The incubation was performed with eluates, as well as in direct contact to scaffolds.

Human osteosarcoma cells (SaOS-2) originating from the cell line of a human osteosarcoma (European Collection of Cell Cultures, No. 89050205, Salisbury, United Kingdom) were used for the experiments. After washing in PBS (pH: 7.4), the cells were seeded as explants into the culture flasks and cultivated at 37 °C in a humidified atmosphere of 95% air and 5% CO_2_. Culture medium was DMEM, supplemented with fetal calf serum, 1% Penicillin/Streptomycin, 2 mM L-glutamine, 100 nM dexamethasone, and 1 mM L-ascorbic acid 2-phosphatase (Sigma-Aldrich, St. Louis, MO, USA). Cells were subcultivated in a second passage at a density of 3.3 × 10^6^/cm^2^. Cell dispenser was used to bring the cells into suspension for the second passage. One hundred microliters containing 3.3 × 10^5^ SaOS-2 cells in the second passage were transferred onto the samples.

In order to comparatively assess both cell viability and cell proliferation, cells were additionally seeded on bare glass as a control. The mean optical density on bare glass at the corresponding interval has been set as 100% and the results of 3-(4,5-dimethylthiazol-2-yl)-2,5-diphenyl-2H-tetrazolium bromide (MTT) and Bromodeoxyuridine (BrdU) assays were calculated accordingly in percentages.

### 2.6. Analysis of Cell Viability—MTT Assay

To assess the viability of HOBs and SaOS-2 cells on PLA (10 mm × 10 mm × 1 mm), SVP-PLA (10 mm × 10 mm × 1 mm) and glass (r: 2.5 mm × 1 mm), am MTT assay was conducted at 6, 24, and 72 h. Briefly, 96-well microtiter plates with 5 × 10^3^ cells per well were incubated and a sample of 100 μL eluate was obtained to identify the metabolically active cells via in vitro Cell Proliferation KIT 1 (Roche, 11465007001, Mannheim, Germany). The optical density of the samples was performed photometrically at 450 nm wavelength.

### 2.7. Analysis of Cell Proliferation—5-Bromo-2′-deoxyuridine (BrdU) Assay

Similar to the MTT processing, proliferating HOBs and SaOS-2 cells on PLA, SVP-PLA, and glass (r: 2.5 mm × 1 mm) were identified via the BrdU (Bromdesoxyuridin) Cell Proliferation ELISA (enzyme linked immune-adsorption) kit (Roche Diagnostics, Mannheim, Germany). Briefly, 96-well microtiter plates with a density of 5 × 10^3^ cells per well were incubated, and a sample of 150 μL eluate was obtained. Calibration curves of 0.16–10 × 10^3^ cells/well served as standards. The optical density of the individual samples was measured via microplate reader (Spectra Max plus 384, Molecular Devices, Sunnyvale, CA, USA) photometrically at 450 nm wavelength.

### 2.8. Immunostaining and Florescence Imaging

The viability of the cells was identified by staining with fluorescein diacetate (FDA, Sigma-Aldrich, St. Louis, MO, USA) and propidium iodide (PI, Sigma-Aldrich, St. Louis, MO, USA). Eight-well plates with 1 × 10^4^ cells per each well were supplied with standard nutrient medium. The cells were washed with PBS and stained with the FDA solution containing 30 μL of stock solution (1 mg FDA/mL acetone) diluted in 10 mL PBS. After an incubation period of 15 min at 37 °C in the dark environment, the FDA solution was removed and replaced by 500 μL PI stock solution. After an incubation time of 120 s, the cells were then washed twice with PBS. Within 60 min after staining, the cells were examined via a fluorescence microscope (Axioplan2) and documented with a digital camera (AxioCam MRc5 from ZEISS, Oberkochen, Germany). The dyes could be excited at 488 nm (blue light, argon laser). The green fluorescence (FDA) was detected at 530 nm, whereas the red fluorescence (PI) was detected at 620 nm.

### 2.9. Cell Morphology Analysis

The morphology of both cell groups was examined by using SEM (XL30CP, Philips Electron Optics GmbH, Kassel, Germany). Following the removal of the cell medium, cell medium, fixation with glutaraldehyde 3% in PBS was carried out at a pH value of 7.4 for 24 h. After removal of the glutaraldehyde solution, the cells were dehydrated in an ascending alcohol dilution for 300 s for each series. After that, drying with hexamethyldisilane for 1 min (Sigma-Aldrich, St. Louis, MO, USA) and a gold vapor deposition with the thickness of 15 nm (SCD 500, CAL-Tec, Ashford, UK) were performed and SEM analysis was conducted at a voltage between 10–15 kV.

### 2.10. Statistical Analysis

All experiments were performed in triplicate, and results were reported as the mean ± SD. Data between three or more groups were compared using the one-way analysis of variance, followed by the Dunnett’s post hoc test. The significance level was set to *p* < 0.05.

## 3. Results and Discussions

### 3.1. Material Characteristics

**Figure 2:**
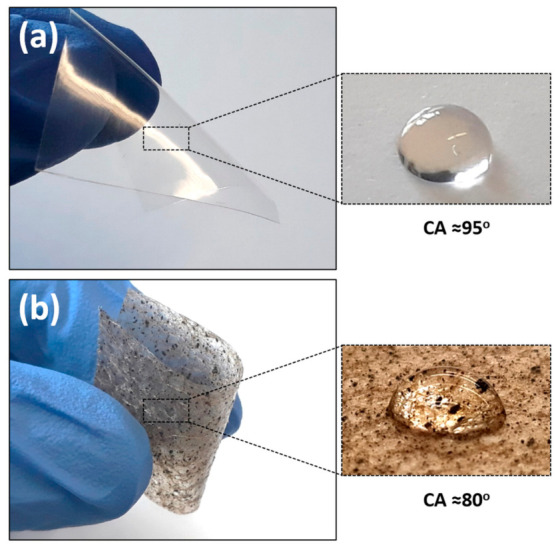
(**a**) Neat PLA (**b**) SVP-PLA composite patch and corresponding water contact angle (WCA) results.

A digital camera was used to visualize the macroscale properties of the prepared neat PLA and SVP-PLA composite patches. Basically, neat PLA and SVP-PLA could exhibit a proper elasticity, which is could be crucial for the treatment of bone defects with complex geometries (Figure 2). Both neat PLA (Figure S1) and SVP-PLA patches exhibit a similar morphology composed of macroscale pores and microscale pin-lock type defects (Figure 2b). While the pore size of samples ranges from 250 µm to 400 μm, the size of pin-locks is around 30–40 µm [26]. On the other hand, on porous SVP-PLA composite patches, brownish residuals were also detected, which were not present on neat PLA. This might be explained by due agglomeration of algae powders, which seem to alter the surface energy since the water contact angle (WCA) of SVP-PLA is lower than that of neat PLA. Definitely, the porous morphology of the patch surface also plays a major role for wetting behavior. For instance, Liu et al. showed that the WCA of pristine PLA was 129.2° when it is prepared in the form of randomly grown nanofibers [30]. The Cassie–Baxter model suggests that an indistinctly hydrophobic material gets more hydrophobic with the increasing roughness [31].

**Figure 3:**
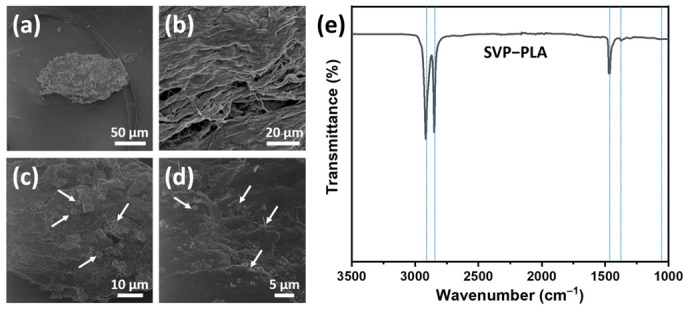
(**a**–**d**) Helium ion microscopy (HIM) images and (**e**) FTIR spectra of the SVP-PLA composite.

A typical algae particle embedded in PLA matrix is shown with an HIM micrograph image in Figure 3a. At higher magnification, it is clearly seen that the particle exhibits a flake-like layered morphology (Figure 3b). Figure 3c,d show that there are crystal-like structures (indicated by arrows) at the top of flake-like structures, which is typical for algae particles [11]. HIM micrograph indicates a poor interfacial adhesion between SVPs and the PLA matrix. Such a poor adhesion was also reported in similar types of composites and attributed to large differences in the surface energies between algae powders and the polymer matrix [11].

FT-IR spectra were recorded for SVP-PLA composite, as shown in Figure 3e. Five peaks at 2915, 2845, 1467, 1374, and 1060 cm^−1^ were identified in the FTIR spectra of SVP-PLA composite. Two characteristic peaks at 2915 and 2848 are assigned to a C—H stretching vibration [32]. The asymmetric and symmetric bending absorption of CH_3_ can be assigned at consecutive peaks at 1467 and 1374 cm^−1^, respectively [33]. These peaks are all typical and indicative of the identification of PLA. The C—O—C vibrations of cellulose, arising from SVP, might be assigned by broadening peak around 1050 cm^−1^ [34]. One should keep in mind that since the 80% of the SVP-PLA composite is made of PLA it is difficult to resolve specific peaks corresponding SVP. However, all peaks can be identified with individual materials in the SVP-PLA composite as shown in Figure S2.

### 3.2. Cell Viability and Proliferation

**Figure 4:**
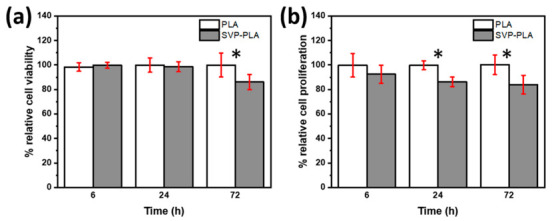
(**a**) MTT and (**b**) BrdU assay of human osteosarcoma (SaOS-2) cells (* represents the statistical significance between subgroups).

MTT assay (Figure 4a) shows that SaOS-2 cells cultured on SVP-PLA exhibited a significant decrease in cell viability in comparison to HOBs with extended cell culturing time (from 6 h to 72 h). While SaOS-2 cells cultured on both neat PLA and SVP-PLA exhibited a slight superiority cell viability of PLA at examination periods of 6 h (p:0.437) and 24 h (0.665), the difference became meaningful at longer periods (72 h, p:0.015).

The BrdU assay shows the SVP-PLA patch induced a decrease in SaOS-2 cell proliferation during the whole cell culturing period significantly, as shown in Figure 4b (p: 0.173, p:0.000 and p:0.005 at 6, 24 and 72 h respectively). Since nutrients in the cell medium were consumed by the time overall cell proliferation decreased at longer cell culturing periods. Nevertheless, there is a clear difference between the proliferation of SaOS-2 cells cultured on neat PLA and SVP-PLA.

**Figure 5:**
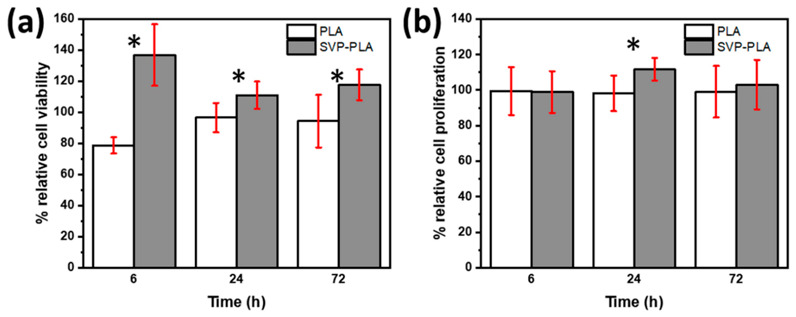
(**a**) MTT and (**b**) BrdU assay of human osteoblast (HOB) cells (* represents the statistical significance between subgroups).

The MTT assay (Figure 4a) showed that SaOS-2 cells cultured on SVP-PLA exhibited a significant decrease in cell viability in comparison to HOBs with extended cell culturing time (from 6 h to 72 h). While SaOS-2 cells cultured on both neat PLA and SVP-PLA exhibited a slightly superior cell viability of PLA at examination periods of 6 h (p:0.437) and 24 h (0.665), the difference became meaningful at longer periods (72 h, p:0.015). The BrdU assay shows the SVP-PLA patch induced a decrease in SaOS-2 cell proliferation during the whole cell culturing period significantly, as shown in Figure 4b (p: 0.173, p:0.000 and p:0.005 at 6, 24 and 72 h respectively). In contrast to SaOS-2 cells, HOBs cultured on SVP-PLA showed significantly higher cell viability during the whole cell culturing period (6 h–72 h) as shown in Figure 5a (p: 0.002, p:0.021 and p:0.006 at 6, 24 and 72 h respectively). At early stages (6 h) where the cell medium was rich in nutrients, a higher significance between the viability of HOBs cultured on neat PLA and SVP-PLA was observed. The BrdU assay (Figure 5b) was also in agreement with the MTT assay, indicating a proliferative effect of SVP-PLA on HOBs. However, a significant difference could be observed only at the 24 h examination (p: 0.019). Overall, the cell proliferation decreased by the time as an expected consequence of the decrease of nutrients in the cell medium. However, during the whole examination period, HOBs cultured on SVP-PLA showed higher proliferation.

In contrast to SaOS-2 cells, HOBs cultured on SVP-PLA showed significantly higher cell viability during the whole cell culturing period as shown in Figure 5a (p: 0.002, p:0.021 and p:0.006 at 6, 24 and 72 h respectively). At early stages (6 h) where the cell medium was rich with nutrients, we observed a higher significance between the viability of HOBs cultured on neat PLA and SVP-PLA. BrdU assay (Figure 5b) was also in agreement with MTT assay indicating proliferative effect of SVP-PLA on HOBs. However, a significant difference could be observed only at the 24 h examination (p: 0.019). Overall, the cell proliferation decreased by the time as an expected consequence of the decrease of nutrients in the cell medium. However, during the whole examination period, HOBs cultured on SVP-PLA showed higher proliferation.

Although more detailed analysis is needed, the SVP-PLA composite patch seems to exhibit a selective inhibitory effect on cell viability and proliferation for SaOS-2 cells. This may lead to an effective antitumoral effect, without disturbing the cytocompatibility or proliferation of healthy cells of the tumor environment [35].

### 3.3. Immunostaining and Fluorescence Imaging

**Figure 6:**
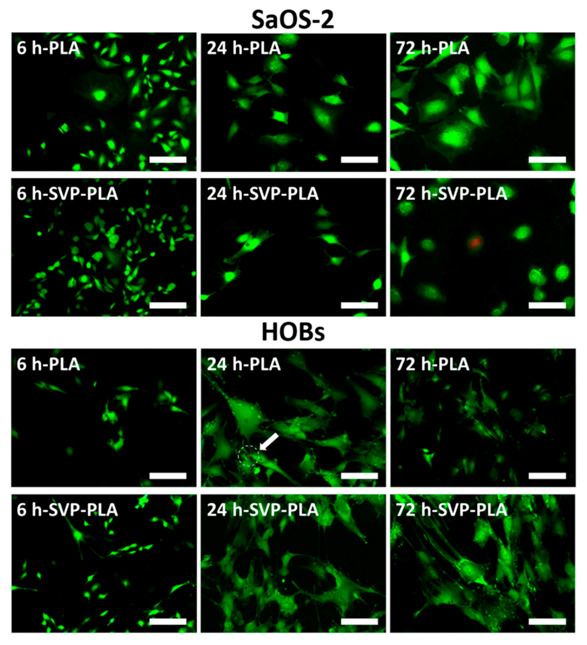
Fluorescence imaging of SaOS-2 cells and HOBs on neat PLA and SVP-PLA composite at 6 h, 24 h, and 72 h culturing time (Scale bars: 200 µm). The results have shown in general lower density of SaSO-2 cells on SVP-PLA patches as compared to the PLA group, which also decreases over time. Besides that, scattered dead bacteria were present on SVP-PLA patches after 72 h. In contrast, a shift to a higher viability of osteoblasts with time could be seen in the SVP-PLA group.

Fluorescence images recorded at an examination period of 6 h indicated that SaOS-2 cells cultured on neat PLA and SVP-PLA had comparable cell densities (Figure 6). However, after 24 h a slight distortion of the cell membrane could be seen in SaOS-2 cells cultured on SVP-PLA. While SaOS-2 cells cultured on neat PLA have maintained their filopodia-like extensions and connected with other cells at 24 h, those cultured on an SVP-PLA patch showed neither filopodia formation nor cell-to-cell interaction. Additionally, nonvital cells could also be detected in the case of SVP-PLA patch. Overall, SAOS-2 cells presented a well-spread morphology on PLA, whereas the cells on SVP-PLA showed a more distorted geometry (Figure 6).

Fluorescence images of HOBs cultured on SVP-PLA present a slight increase in cell density in comparison to those cultured on neat PLA with the time. After 6 h, the filopodia were also more prominent on an SVP-PLA patch. At 24 h and 72 h, HOBs cultured on SVP-PLA maintained their filopodia-like extensions and connected with other cells, whereas those cultured on neat PLA showed significantly less cell-to-cell contact. Additionally, sporadic nonvital cells could also be detected on neat PLA (shown by arrow). Overall, HOBs presented a well-spread morphology on SVP-PLA, whereas they exhibited a slightly distorted geometry on neat PLA (Figure 6).

### 3.4. Morphological Analysis of Cells

**Figure 7:**
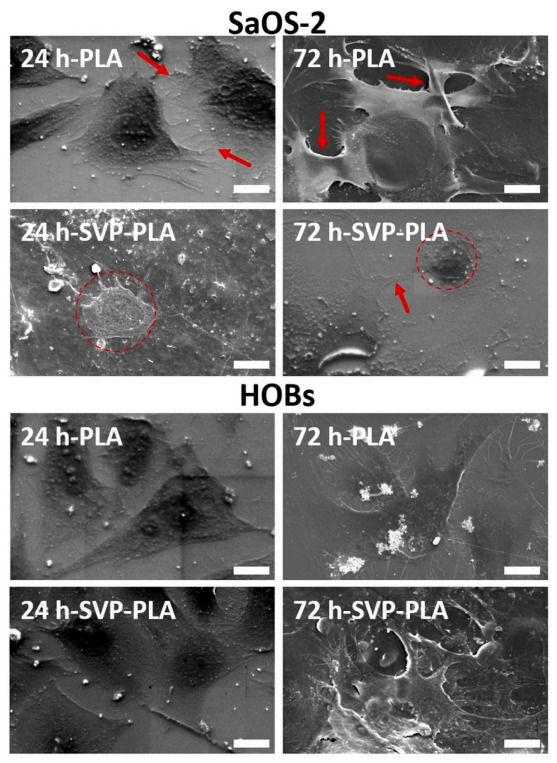
SEM images of SaOS-2 cells and HOBs on neat PLA and SVP-PLA composite at 24 h and 72 h (Scale bars: 20 µm). After 72 h, the filopodia formation was followed by a strong cell–cell connection (shown by red arrows) on a neat PLA patch. (Magnification: 2000×).

SEM analysis showed that, SaOS-2 cells were irregular in shape and the distortion of the cell membrane could be detected after 24 h on SVP-PLA patch (Figure 7). In contrast, SaOS-2 cells exhibited a higher level of filopodia (shown by red arrows) formation on neat PLA, which indicates a feasible cell–surface interaction.

After 72 h, the filopodia formation was followed by a strong cell–cell connection (shown by red arrows) on a neat PLA patch. These morphological findings are in accordance with those observed in the florescence imaging (Figure 7). Clearly SAOS-2 cells cultured on neat PLA seem to spread well and to be viable. On the other hand, we observed an abnormal morphology (indicated by dashed red circle) in SaOS-2 cells cultured on SVP-PLA patch after 12 h. After 72 h examination, although some filopodia formation (shown by red arrow) was observed and SaOS-2 cells seemed to prefer staying isolated (indicated by dashed red circle), rather than forming dense cell–cell contacts as observed in case of neat PLA, on SVP-PLA patch.

SEM analysis of HOBs revealed that filopodia-like subcellular structures could be detected on both neat PLA and SVP-PLA patches at 24 h. However, cell density was on SVP-PLA patches relatively high. At 72 h, HOBs on SVP-PLA showed higher number of filopodia and concomitant interactions, which ensured a clear cell-to-cell contact. These findings are in accordance with morphologies detected in florescence imaging (Figure 6).

Current ex vivo study basically showed that the bifunctional patch described herein could be a promising material in dentistry and orthopedics. PLA is a most commonly used biodegradable material in clinical applications, especially in the field of dentistry and traumatology. However, biodegradation characteristics and especially dissolved organic carbon release is also an important aspect in the context of algae degradation, which should be further studied by in vivo models [36]. Similarly, the clinical efficiency of the bifunctional patch described herein might differ from its composition. Therefore, furthers studies focusing on the dose dependent effects could be beneficial to gain insights into the possible clinical results.

It is well known that algae growing in metal-polluted environments could have a toxicological content. Several studies focusing on the chemical composition of the Sargassum species showed also their possible heavy metal content [37]. Therefore, further studies conducted with novel detection frameworks are needed to clarify the heavy metal toxicity properties of the presented biomaterial herein [38].

## 4. Conclusions

Considering the results expressed herein, it can be concluded that SVP-PLA composite patches may be used to suppress the proliferation of SaOS-2 cells, which could be attributed to the disruption of filopodia between cells, resulting in a lack of cell-to-cell contact. However, the exact mechanism underlying this phenomenon warrants further research. Since the antiproliferative effect of SVP-PLA for SaOS-2 cells does not impair HOB proliferation, it can act as an ideal patch in the management of osseous carcinomas.

## Data Availability

The data presented in this study are available on request from the corresponding author.

## Supplementary Materials

The following are available online at https://www.mdpi.com/2073-4360/13/6/847/s1, Figure S1: SEM images of PLA at different magnifications; Figure S2: FTIR spectra for SVP and PLA.
