# Supplementary material for: Marine Algae Incorporated Polylactide Acid Patch: Novel Candidate for Targeting Osteosarcoma Cells without Impairing the Osteoblastic Proliferation
Source: Polymers (Basel). 2021 Mar 10;13(6):847. doi: 10.3390/polym13060847 (PMC8001715; doi:10.3390/polym13060847)
Supplement: Supplementary file 1 [file polymers-13-00847-s001.pdf]

SUPPLEMENTARY MATERIALS

Marine algae incorporated polylactide acid patch: Novel candidate for targeting osteosarcoma cells without impairing the osteoblastic proliferation

Salih Veziroglu,[1‡] Mustafa Ayna,[2‡] Theresa Kohlhaas,[3‡] Selin Sayin,[4] Jacek Fiutowski,[5] Yogendra Kumar Mishra,[5] Fatih Karayürek,[6] Hendrik Naujokat,[3] Eyüp Ilker Saygili,[7] Yahya Açil,[3] Jörg Wiltfang,[3] Franz Faupel,[1] Oral Cenk Aktas,[1*] Aydin Gülses.[3*]

1 Chair for Multicomponent Materials, Institute for Materials Science, Faculty of Engineering, Kiel University, Kaiserstr. 2, 24143 Kiel, Germany; sve@tf.uni-kiel.de (S.V.); ff@tf.uni-kiel.de (F.F.)
2 Department of Periodontology, University Hospital of Bonn, D-53111 Bonn , Germany; mayna@uni-bonn.de (M.A.)
3 Department of Oral and Maxillofacial Surgery, University Hospital of Schleswig-Holstein, Campus Kiel, Arnold-Heller-Straße 3, 24105 Kiel, Germany; tete@theresakohlhaas.de (T.K.); Hendrik.Naujokat@uksh.de (H.N.); Yahya.Acil@uksh.de (Y.A.); Joerg.Wiltfang@uksh.de (J.W.)
4 Marine Science and Technology Faculty, Iskenderun Technical University, 31200 Iskenderun, Turkey; selin.sayin@iste.edu.tr (S.S.)
5 Mads Clausen Institute, NanoSYD, University of Southern Denmark, Alsion 2, 6400 Sønderborg, Denmark; fiutowski@mci.sdu.dk (J.F.); mishra@mci.sdu.dk (Y.K.M.)
6 Department of Periodontology, Cankiri Karatekin University, 18100 Cankiri, Turkey; fatihkarayurek@karatekin.edu.tr (F.K.)
7 Department of Medical Biochemistry, SANKO University, Şehitkamil, 27090 Gaziantep, Turkey; ISaygili@sanko.edu.tr (E.I.S.)
* Correspondence: oca@tf.uni-kiel.de (O.C.A.); aguelses@mkg.uni-kiel.de (A.G.)
‡ These authors contributed equally.

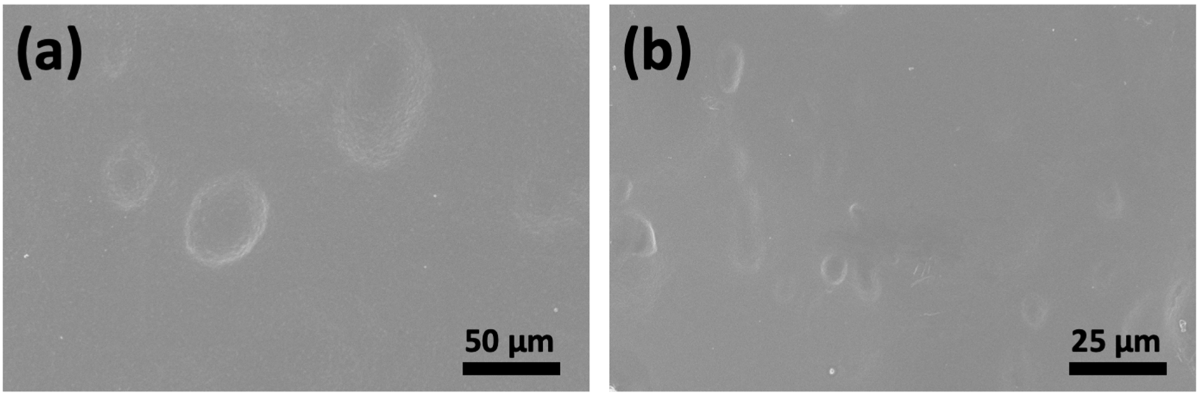

Figure S1. SEM images of PLA patch at different magnifications.

The FT-IR spectrum of Sargassium vulgare and PLA is shown in **Figure** S2. -OH stretching vibrations in the PLA structure come as a wide band of 3354 cm^{-1}. -CH_3 stress vibrations were observed around 2955 cm^{-1}, 2946 cm^{-1}. It was observed that the absorption of C=O stretching vibrations was came at 1704 cm^{-1}, and

C=O asymmetric bending adsorption at 1454 cm^{-1}. CH and CH_3 symmetric bending were 1368, 1319 cm^{-1}, and the asymmetric stress vibrations of CH_3 come around 1406 cm^{-1}. C-O-C stress absorptions of 1242, 1155, 1142, and 1052 cm^{-1} were observed due to the different sequences of the C-O-C function groups. It was observed that the O-CH-CH_3 band absorption was 882 cm^{-1} and the bending absorption of -CH_3 came at 775 cm^{-1}. The wide -OH absorption band coming around 3500 cm^{-1}, the C=O band absorption at 1704 cm^{-1}, and the bending absorption band of -CH_3 at 775 cm^{-1} were defined the structure of the PLA [1].

It was caused by the -OH stress vibrations in the 3300 cm^{-1} wide-bandgap structure belonging to sargassum Vulgaris, which is marina algae. O-C-O symmetrical band stress vibrations in the polysaccharide ring were seen at 1410 cm^{-1}. C-C-H and C-O band tensile vibrations were observed to come at 1018 and 873 cm^{-1} [2].

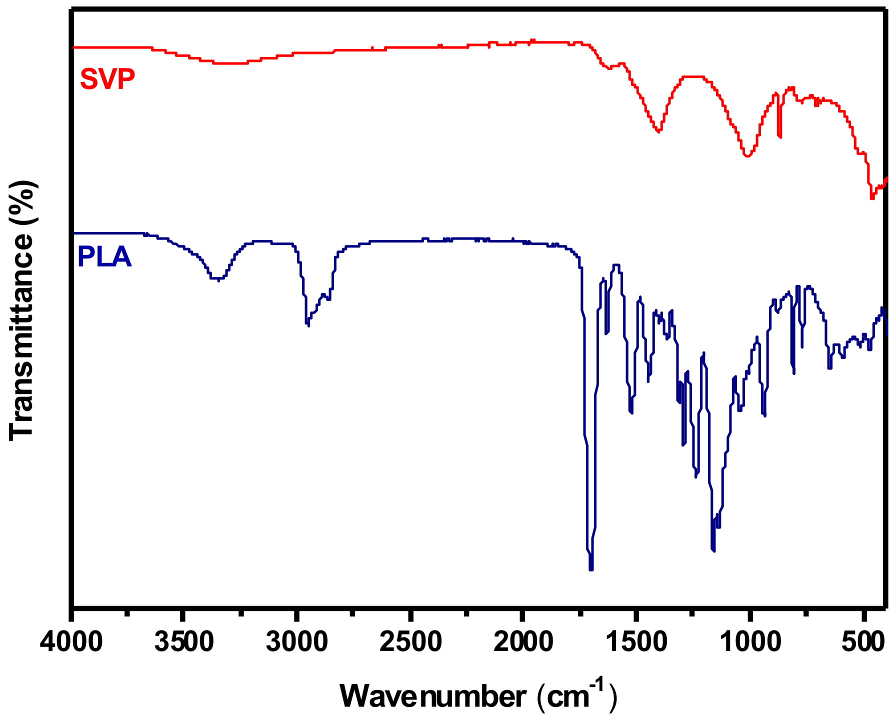

Figure S2. FTIR spectra for SVP and PLA

References

1. Cai, Y., Lv, J. & Feng, J. Spectral Characterization of Four Kinds of Biodegradable Plastics: Poly (Lactic Acid), Poly (Butylenes Adipate-Co-Terephthalate), Poly (Hydroxybutyrate-Co-Hydroxyvalerate) and Poly (Butylenes Succinate) with FTIR and Raman Spectroscopy. J Polym Environ 21, 108–114 (2013).

2. Dalia M.S.A. Salem, Mona M. Ismail, Hermine R.Z. Tadros, Evaluation of the antibiofilm activity of three seaweed species and their biosynthesized iron oxide nanoparticles (Fe_3O_4-NPs), The Egyptian Journal of Aquatic Research, Volume 46, Issue 4, 2020, Pages 333-339
